# Research on the Performance of Thermoelectric Self−Powered Systems for Wireless Sensor Based on Industrial Waste Heat

**DOI:** 10.3390/s24185983

**Published:** 2024-09-15

**Authors:** Yong Jiang, Yupeng Wang, Junhao Yan, Limei Shen, Jiang Qin

**Affiliations:** 1 School of Energy and Power Engineering, Huazhong University of Science and Technology, Wuhan 430074, China; m202171148@alumni.hust.edu.cn (Y.J.); 12131019@mail.sustech.edu.cn (Y.W.); d202480537@hust.edu.cn (J.Y.); 2 Department of Materials Science and Engineering, Southern University of Science and Technology, Shenzhen 518055, China; 3 School of Energy Science and Engineering, Harbin Institute of Technology, Harbin 150001, China; qinjiang@hit.edu.cn

**Keywords:** thermoelectric generator, wireless sensor, power management integrated circuit, MPPT optimization, industrial waste heat

## Abstract

The issue of energy supply for wireless sensors is becoming increasingly severe with the advancement of the Fourth Industrial Revolution. Thus, this paper proposed a thermoelectric self−powered wireless sensor that can harvest industrial waste heat for self−powered operations. The results show that this self−powered wireless sensor can operate stably under the data transmission cycle of 39.38 s when the heat source temperature is 70 °C. Only 19.57% of electricity generated by a thermoelectric power generation system (TPGS) is available for use. Before this, the power consumption of this wireless sensor had been accurately measured, which is 326 mW in 0.08 s active mode and 5.45 μW in dormant mode. Then, the verified simulation model was established and used to investigate the generation performance of the TPGS under the Dirichlet, Neumann, and Robin boundary conditions. The minimum demand for a heat source is cleared for various data transmission cycles of wireless sensors. Low−temperature industrial waste heat is enough to drive the wireless sensor with a data transmission cycle of 30 s. Subsequently, the economic benefit of the thermoelectric self−powered system was also analyzed. The cost of one thermoelectric self−powered system is EUR 9.1, only 42% of the high−performance battery cost. Finally, the SEPIC converter model was established to conduct MPPT optimization for the TEG module and the output power can increase by up to approximately 47%. This thermoelectric self−powered wireless sensor can accelerate the process of achieving energy independence for wireless sensors and promote the Fourth Industrial Revolution.

## 1. Introduction

The Internet of Things (IoT) is a network concept that enables users to control things easily through the Internet. Along with the Fourth Industrial Revolution, the IoT has been growing rapidly [1], especially in the fields of civil infrastructure [2], environmental monitoring [3], and intelligent manufacturing [4,5]. Forecasts report that, by 2027, there will likely be more than 29 billion IoT connections worldwide [6]. The wireless sensor, designed for information perception and data acquisition, is the main component of the IoT, and also its “nerve ending” [7]. Nowadays, wireless sensors are primarily powered by batteries. However, batteries [8], with their finite energy capacity, require frequent recharging or replacement, leading to significant inconvenience and considerable expense. This issue will become particularly critical when a multitude of wireless sensors are deployed in remote locations. Moreover, the presence of hazardous chemicals in batteries raises concerns about environmental contamination. Therefore, the energy issue emerges as a significant constraint for the development of wireless sensors.

Despite the generally low power consumption of individual wireless sensors, when deployed on a massive scale and required to operate stably over long periods, they will lead to significant energy consumption, which in turn results in substantial carbon emissions. Therefore, in line with the global trend towards carbon neutrality, to tackle the energy supply challenges faced by wireless sensors, many studies have proposed the use of self−powered systems that can harvest energy from the environment surrounding the wireless sensors [9], such as solar energy [10], wind energy [11], and mechanical energy [12], thereby realizing achieving energy independence for wireless sensors. However, the majority of renewable energy sources are variable and cannot consistently supply power around the clock. Thus, they cannot be employed to produce electricity continuously [13]. In recent years, the thermoelectric generator (TEG) has gradually entered people’s view as an alternative solution to the power supply challenge for wireless sensors [14,15,16], as it can directly convert heat into electricity via the Seebeck effect [17,18]. TEGs have the advantages of having no moving parts, providing environmental protection, long lifetime, high reliability, and low maintenance costs [19,20]. Moreover, they can realize stable power generation when there exist continuous heat sources. At present, a large part of China’s industrial energy consumption is directly discharged into the environment as heat [21], which is also known as industrial waste heat. According to the temperature range, industrial waste heat can be divided into three grades: high−temperature industrial waste heat above 500 °C, medium−temperature industrial waste heat between 200 °C and 500 °C, and low−temperature industrial waste heat below 200 °C [22]. Taking China’s iron and steel industry as an example, the proportion of high, medium, and low−temperature industrial waste heat generated in the production process is, respectively, 39.8%, 25.9%, and 34.2% [23]. If this industrial waste heat can be utilized by a TEG to power wireless sensors, it would not only enhance energy utilization efficiency and contribute to carbon neutrality but also play a significant role in promoting the development of the Fourth Industrial Revolution.

To date, many studies have been conducted on the thermoelectric self−powered system based on wireless sensors. As early as 2006, Jovanovic et al. [24] designed and fabricated a prototype of a thermoelectric self−powered system. Nevertheless, this system did not consider the circuit matching problem between the self−powered system and the sensor. Given that the output voltage of TEG is typically low and subject to fluctuations with external temperature differences, and wireless sensors require a stable input voltage for power supply, an external step−up converter and a power management integrated circuit (PMIC) are necessary between the TEG and wireless sensors. Therefore, subsequent research has almost universally involved the design of relevant PMIC. For instance, Wang et al. [25] developed a two−stage ultra−low voltage step−up converter for a thermoelectric self−powered system, achieving an overall circuit efficiency of up to 25.2%. They also emphasized that an accurate analysis of wireless sensor power consumption is an essential part of the design process for thermoelectric self−powered systems. However, many current studies have fallen short in this regard. For example, Wang et al. [26] proposed a thermoelectric self−powered system based on wireless sensors for aircraft health monitoring, and Li et al. [27] concentrated on a thermoelectric self−powered battery activated by temperature changes for forest fire alarms. They both focused mainly on optimizing the power generation performance of TEG, while completely neglecting the power consumption testing of the wireless sensors. Additionally, some studies [28,29] directly used power consumption data sheets provided by vendors, which is also unacceptable. This is because wireless sensors may be configured with different data transmission cycles during actual operation, leading to varying power consumption.

Furthermore, accurately assessing the circuit efficiency of power management integrated circuits is essential. This will ensure precise alignment between the energy supply of thermoelectric self−powered systems and the energy demands of wireless sensors, and can guide the design of suitable thermoelectric self−powered systems. Regrettably, numerous studies [30,31,32,33] have overlooked this critical research focus. Although these studies enabled wireless sensors to be powered by thermoelectric self−powered systems, they cannot ensure that the electric energy generated is fully utilized, potentially leading to energy waste. Only a mere handful of studies focused on both circuit efficiency and the matching of energy supply and demand. For instance, An et al. [34] proposed a thermoelectric self−powered system that can harvest body heat to fully power wireless sensors, which integrated the LTC3108 chip for power management. This study showed that the TEG must generate 73 μW of electrical power to offset the 29 μW consumed by the wireless sensor, indicating an energy loss due to the LTC3108’s conversion efficiency of less than 50%.

It is acknowledged that TEG can achieve maximum output power when external load equals its internal resistance. However, in practical applications, the internal resistance of TEG tends to vary with changes in external temperature, making it difficult to operate stably at the maximum power point (MPP). Unfortunately, most of the aforementioned studies cannot guarantee TEG operation at the MPP. Only the studies by Li et al. [27] and Kim et al. [28] employed a PMIC based on the BQ25504 chip, which incorporates the Maximum Power Point Tracking (MPPT) capability. However, they did not elucidate the details of MPPT optimization or demonstrate the benefits of MPPT. Moreover, many commercially available power management integrated circuits lack MPPT technology. Consequently, it is crucial to optimize the output power of thermoelectric self−powered systems based on PMIC, because it can markedly improve the system’s conversion efficiency [35]. As an example, Guan et al. [36] developed a two−stage step−up converter for a thermoelectric self−powered system, where the first stage includes the MPPT function. They found that when the TEG’s open circuit voltage ranged from 84 mV to 400 mV, the circuit’s conversion efficiency was between 44.2% and 75.4%, outperforming the commercially available converter based on the BQ25504 chip. Moreover, relevant studies have specifically conducted MPPT optimization of TEG’s output power based on SEPIC converter [37], Cuk converter [38], and Boost converter [39]. Among these, charging a battery through a TEG with a SEPIC converter featuring MPPT technology is 15% more efficient than charging the battery directly with the TEG, and the TEG’s output power, when processed by a Cuk converter featuring MPPT technology, is 14.5% higher than without MPPT technology and 22.6% higher than direct charging.

Combining the aforementioned studies, it can be observed that the complete process of designing a thermoelectric self−powered system based on wireless sensors should encompass the following steps: accurate analysis of the wireless sensor’s power consumption, design and efficiency evaluation of the PMIC, structural design and performance characterization of the thermoelectric self−powered system, and MPPT optimization for TEG based on PMIC. However, previous studies have either focused solely on optimizing the TEG’s power generation performance for specific applications or specifically on MPPT optimization for TEG’s output power. There is a scarcity of studies that integrate these two aspects to form a comprehensive study spanning the spectrum from design to optimization of thermoelectric self−powered systems. The majority of research has overlooked the accurate analysis of the power consumption for wireless sensors, which is a critical foundation for the design of practical thermoelectric self−powered systems and cannot be disregarded. Furthermore, although the possibility of thermoelectric self−powered sensors has been thoroughly verified by current studies, there is still no commercial thermoelectric self−powered system with a simple structure and low price for wireless sensors.

Therefore, this paper designed, fabricated, and tested a thermoelectric self−powered system for wireless sensors based on industrial waste heat. Firstly, the wireless sensor’s power consumption and the TEG’s power generation performance were tested through experiments, and a power management integrated circuit was designed based on the LTC3108−1 chip, and its efficiency was also experimentally evaluated. These tests provided data for designing the thermoelectric self−powered system. Subsequently, the thermoelectric self−powered system was designed and its performance was calculated through simulation under different thermal conditions. Then, the actual performance of the thermoelectric self−powered wireless sensor was tested by experiments. Moreover, the economic benefit of this system was also analyzed to investigate its prospects for commercialization. Finally, a SEPIC converter model was developed using the Simulink module within MATLAB R2017a to perform MPPT optimization for the TEG’s output power, and the impact of the MPPT technology on the optimization of thermoelectric self−powered systems was investigated.

## 2. Design of Thermoelectric Self−Powered System

### 2.1. Power Consumption of Wireless Sensor

**Figure 1:**
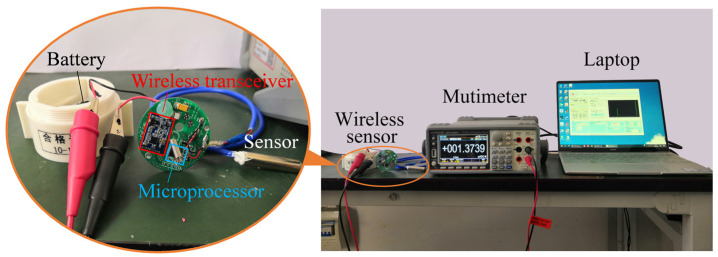
The test rig for testing power consumption of the wireless sensor.

Before designing the thermoelectric self−powered system, a precise analysis of the wireless sensor’s power consumption must be conducted. A commercial temperature wireless sensor (YGWT−2000S/P) was selected to test the average power consumption. It is composed of a temperature sensor, microprocessor, wireless transceiver, and battery. Some important technical specifications are presented in Table 1. A multimeter (GWINSTEK−GDM 9601) was used to measure the real−time current and voltage directly when the wireless sensor is running, as shown in Figure 1.

**Figure 2:**
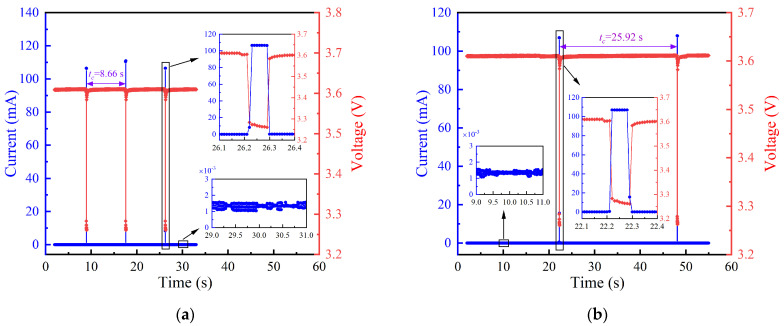
Operating current and voltage of wireless sensor under various data transmission cycles: (**a**) *t_c_* = 8.66 s; (**b**) *t_c_* = 25.92 s.

The data transmission cycle is defined as the interval time between two consecutive data transmissions by the sensor, encompassing the time of dormant mode and data transmission mode. In this section, the data transmission cycles were set to 8.66 s and 25.92 s. Figure 2 depicts the operating current and voltage of the wireless sensor when the data transmission cycle (*t_c_*) is 8.66 s and 25.92 s. It can be seen that the current is tiny under the dormant mode, which is only about 1.41 μA. When the wireless sensor transmits data, the current will suddenly rise to approximately 108 mA, which is much higher than the dormant current. Conversely, the voltage sharply drops from approximately 3.6 V to 3.27 V as the wireless sensor shifts from dormant mode to data transmission mode. Meanwhile, it can be observed from Figure 2 that the data transmission mode lasts about 0.08 s, and the wireless sensor’s energy consumption is primarily concentrated in this phase.

**Figure 3:**
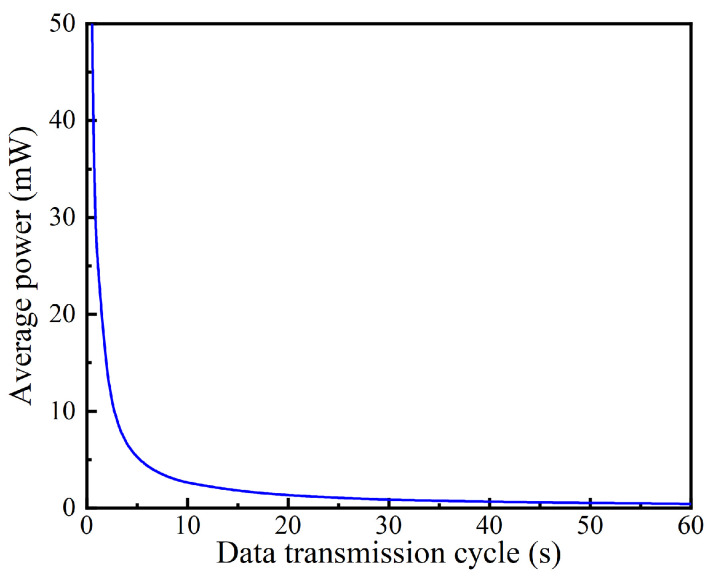
Average power of wireless sensor under various data transmission cycles.

After calculating the overall experimental data, the transmitting power and the dormant power of the wireless sensor are 326 mW and 5.45 μW, respectively. Subsequently, because the time of data transmission mode, the transmitting power, and the dormant power are constant, we can calculate the average power of the wireless sensor under any data transmission cycle. Thus, the average power consumption of the wireless sensor under different data transmission cycles is estimated, as shown in Figure 3. The average power initially decreases rapidly and then decreases slowly with the increase in the data transmission cycle. The average power of the wireless sensor is 26.1 mW for *t_c_* = 1 s, reducing to 5.22 mW for *t_c_* = 5 s, and lower than 1 mW for *t_c_* > 26 s. Thus, it is feasible to power the wireless sensor through a TEG based on industrial waste heat.

### 2.2. Generation Performance of TEG Module

**Figure 4:**
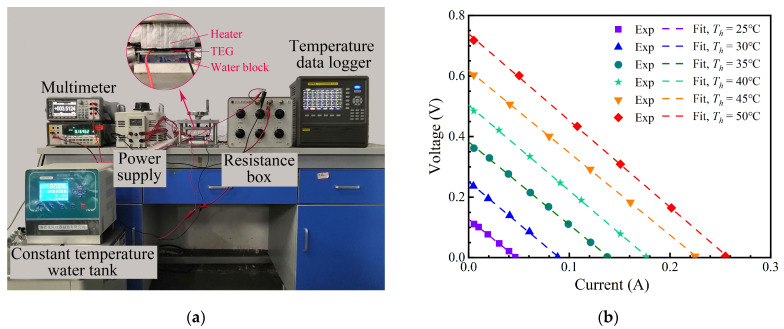
The power generation performance test of TEG module: (**a**) test rig; (**b**) experimental results.

A commercial TEG module (TEG1−127−1.4−1.6−250) is used in this system. The maximum allowable hot side temperature is 250 °C. A test rig was established to measure the generation performance of the TEG module, as shown in Figure 4a. The TEG module was sandwiched between the heat source, provided by a heater, and the cold source, a water block maintained at a fixed temperature. The heater was surrounded by thermal insulation material, and thermal grease was applied to minimize the thermal contact resistance at contact surfaces. The temperature of the TEG module was measured by T−type thermocouples (TT−T−40−SLE−1000) and transmitted to the temperature data logger (TOPRIE TP700). The resistance box was directly connected to the TEG module as the external load. The current and the external load voltage were measured by the digital ammeter (FLUKE 8808A) and digital voltmeter (GWINSTEK−GDM 9601), respectively. It started to record the experimental data when the temperatures reached a stable value. The experiments were carried out by changing the hot side temperature (*T_c_*) from 25 °C to 50 °C with an interval of 5 °C, but maintaining the cold side temperature at 20 °C. Figure 4b describes the variation of voltage versus the current of the TEG module. The internal resistance (*R_in_*) of the TEG module can be estimated according to the slope of the voltage−current curve. Moreover, the open−circuit voltage and maximum output power under different conditions can be derived from Figure 4b and are listed in Table 2. The average internal resistance of the TEG module is 2.77 Ω, with a maximum relative error of 3.5%. The relationship between the maximum output power (*P_max_*) of the open−circuit voltage (*U_oc_*) can be obtained easily as follows.

*P_max_* = 0.09 × *U_oc_*^2^ (1)

### 2.3. Power Management Integrated Circuit of Thermoelectric Self−Powered System

**Figure 5:**
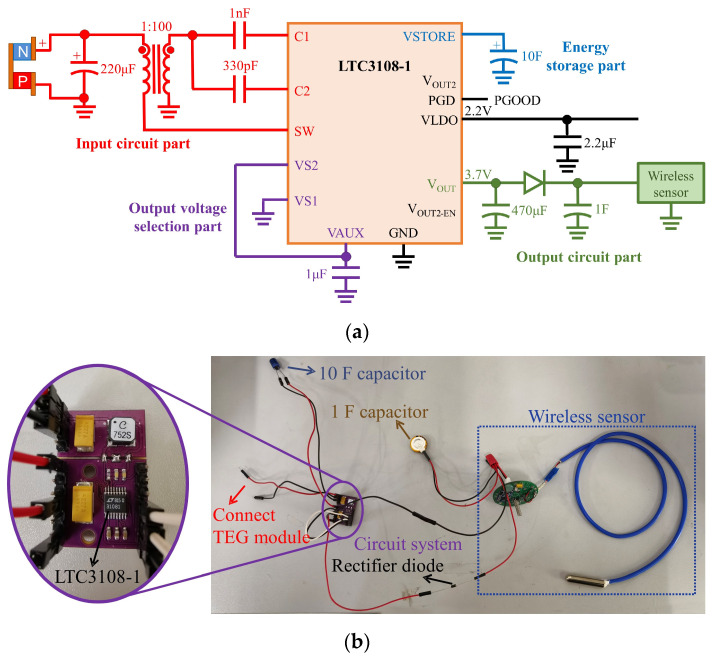
The design and production of power management integrated circuit: (**a**) schematic diagram; (**b**) practical photo.

The power management integrated circuit is a must for the thermoelectric self−powered system because of the low voltage and power fluctuation of the TEG module. The LTC3108−1 produced by the Linear Technology Corporation is a fine choice to collect and manage an overabundance of energy from the low input voltage, as low as 20 mV. The PMIC of the thermoelectric self−powered system based on LTC3108−1 is shown in Figure 5a.

The whole PMIC is divided into four parts: an input circuit part, an output voltage selection part, an energy storage part, and an output circuit part. The input circuit part connects the TEG module with the PMIC, which consists of an external DC−DC step−up converter that can raise the voltage from 20 mV to 500 mV so that this PMIC can harvest ultra−low voltage electricity. The output voltage selection part determines the output voltage of the PMIC. The LTC3108−1 chip offers a choice of four output voltages: 2.5 V, 3 V, 3.7 V, and 4.5 V. The output voltage is set to 3.7 V depending on the voltage requirements of the wireless sensor. A 10 F capacitor is used in the energy storage part to store the surplus electricity for −long−term use. The wireless sensor is directly attached to the output circuit part. Simultaneously, a fully charged 1 F capacitor is also connected to the wireless sensor in parallel to ensure the power consumption required for the wireless sensor startup. A rectifier diode is added to avoid the 1 F capacitor charging to LTC3108−1 when the wireless sensor is not working. The total PMIC is presented in Figure 5b. In this PMIC, all parts of the circuit system are integrated except for two energy storage capacitors and a rectifier diode.

**Figure 6:**
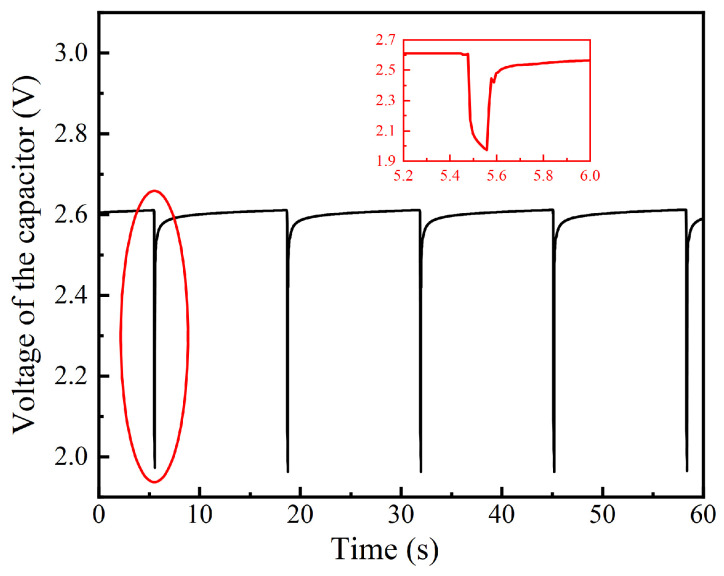
The variation of 1 F capacitor’s voltage.

To test the circuit efficiency of the PMIC, experiments were carried out based on the test rig for testing the power generation of the TEG module. The voltage of the 1 F capacitor and 10 F capacitor were measured by a digital voltmeter (GWINSTEK−GDM 9601) and another digital voltmeter (FLUKE 8808A), respectively. The hot side and cold side temperatures of the TEG module were maintained at 17.5 °C and 30 °C. After the system is stable, the 1 F capacitor’s voltage is exhibited in Figure 6. The data transmission cycle of the wireless sensor is only 13.2 s in this experiment. When the wireless sensor starts transmitting data, the voltage of the 1 F capacitor drops instantaneously from 2.61 V to 1.97 V in 0.08 s and then bounces back to 2.5 V in 0.06 s. As the TEG module charges the capacitor, its voltage slowly retunes to 2.61 V under the dormant state. It should be noted that the voltage of another 10 F capacitor is almost unchanged. Thus, the power consumption of the wireless sensor is equal to the electricity charged by the TEG module. Although the capacitor’s voltage does not reach the voltage of the battery used in the commercial wireless sensor, the wireless sensor can still work, which can be confirmed by observing the aggregation terminal.

The energy consumption of the wireless in one data transmission cycle is 0.026 J according to Section 2.1. The output voltage and current of the TEG module were 251 mV and 83 mA, respectively. The electricity generated by the TEG module is 0.275 J in 13.2 s. Thus, the circuit efficiency is very low, at only 9.45%, which is consistent with the datasheet of LTC3108−1.

### 2.4. Structure of Thermoelectric Power Generation System

**Figure 7:**
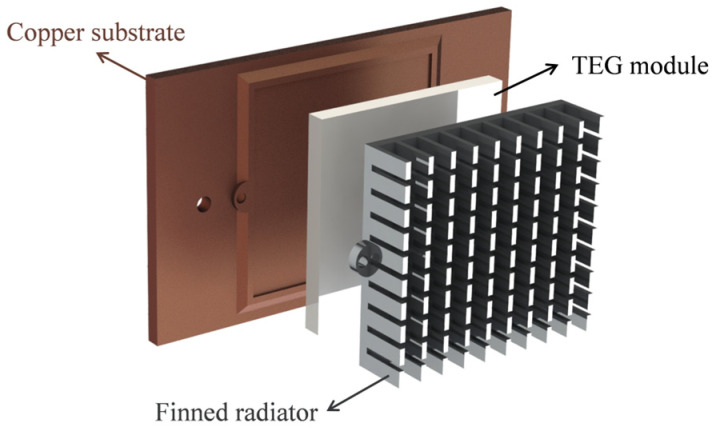
Structure of the thermoelectric power generation system.

According to the above discussion, the power needed from the thermoelectric power generation system (TPGS) and the minimum open−circuit voltage of the TEG module are estimated as the design demands of the TPGS, as listed in Table 3. It should be noted that the circuit efficiency of 9.45% is considered constant. To design a thermoelectric self−powered system with high commercialization potential, the system structure of TPGS is as simple as possible. Thus, the system only consists of three parts: the copper substrate, the TEG module, and the finned radiator, as shown in Figure 7. Natural convection is used to cool the self−powered system because it does not consume electricity. The substrate is used to harvest the heat of the heat source and is made of copper with high thermal conductivity. A 0.5 mm deep groove is provided on the copper substrate for fixing the TEG module. A commercial finned radiator is selected to cool the TEG to establish the temperature difference. The radiator is made of aluminum and is connected to the copper substrate through the plastic screw to reduce the heat loss from the joint. The commercial TEG module (TEG1−127−1.4−1.6−250) is used in this system.

It can be seen that this thermoelectric power generation system has a straightforward structure and is easy to fabricate, which has good commercialization potential. Furthermore, the generation performance of this system would be evaluated accurately in subsequent studies.

## 3. Simulation Analysis of Thermoelectric Power Generation System

### 3.1. Simulation Model and Validation

**Figure 8:**
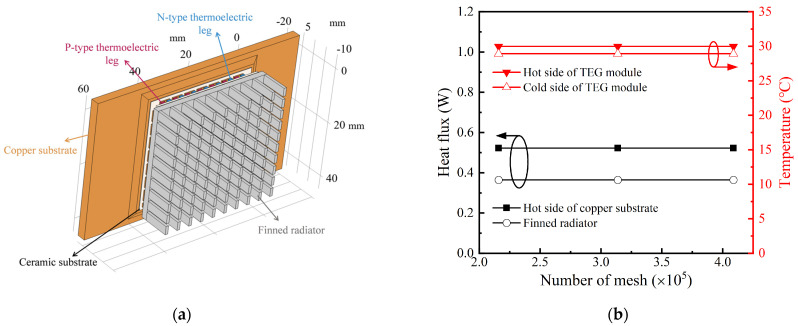
The mesh independence verification of the simulation model: (**a**) geometry parameters of the simulation model; (**b**) mesh independence verification results.

To calculate the generation performance of the TPGS, the simulation model was established, as shown in Figure 8a. A thickness of 0.1 mm was built in the interface as the thermal grease and the contact thermal resistance, and its thermal conductivity was set to 2 W/m/K. The hot side of the copper substrate is set according to the type of the heat source. Thus, it is set to the constant temperature, constant heat flow, and constant heat convection thermal boundary condition according to the type of the heat source. Moreover, the walls of the finned radiator are set to the fixed heat convection boundary. According to the empirical formula for the natural convection heat transfer coefficient on vertical plate surfaces [40], the convective heat transfer coefficient (*h*) varies based on the temperature of the finned radiator, which can be calculated as:

(2) $$\mathrm{Nu}=0.59{\left(\mathrm{Gr}\cdot \mathrm{Pr}\right)}^{0.25}=0.59{\left(\frac{g\beta \Delta TL^{3}}{v^{2}}\cdot \mathrm{Pr}\right)}^{0.25}=\frac{hL}{\lambda }$$

where Nu, Gr, and Pr, respectively, are the Nusselt number, Grashof number, and Prandtl number. *β*, *ν*, and *λ*, respectively, are the volume expansion coefficient, kinematic viscosity, and thermal conductivity of the air. *g* is the gravitational acceleration. Δ*T* is the temperature difference between the finned radiator (*T_f_*) and the ambience (*T_a_*). Moreover, *L* is the characteristic length of the finned radiator.

Due to the complexity of the overall structure, the hybrid mesh is used in this thesis. The cells have a minimum size of 0.1 mm in the contact layer and a maximum of 1.5 mm elsewhere. The total number of mesh is 215,911, with an average mesh quality of 0.726. Figure 8b indicates that the current mesh is fine enough to ensure mesh independence.

**Figure 9:**
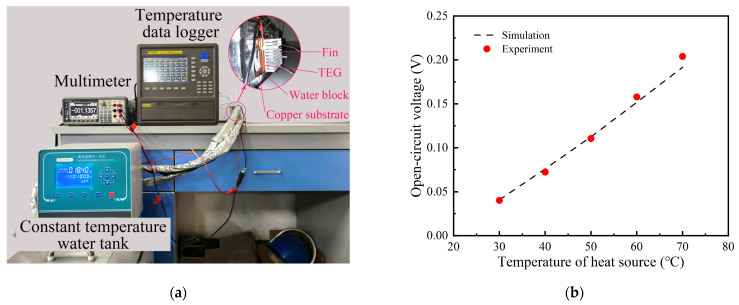
The accuracy validation of the simulation model: (**a**) photo of test rig; (**b**) comparison of open−circuit voltage results.

In order to validate the simulation model and investigate the generation performance of the TPGS, a test rig was built as shown in Figure 9a. A TPGS with the same size as the simulation module was fabricated and heated by the constant temperature water tank. The open−circuit voltage of the TEG module was measured by a multimeter (GWINSTEK−GDM 9601). The temperatures at key locations were tested by T−type thermocouples (TT−T−40−SLE). The experiments were carried out by changing the temperature of the heat source from 30 °C to 70 °C with an interval of 10 °C. At the same time, the boundary conditions of the simulation module were consistent with those of the experiments. The results are shown in Figure 9b. It can also be seen that the simulation results are in good agreement with the experimental results. The errors between simulations and experiments are all less than 10%, which indicates that the numerical model is accurate.

### 3.2. System Performance under Different Heat Sources

To investigate the generation performance of the TPGS, the open−circuit voltage was calculated based on the verified simulation model. The thermal boundary condition, which is related to the practical condition, is a critical parameter for the system application. The thermal boundary condition can be divided into three types: the Dirichlet boundary condition, the Neumann boundary condition, and the Robin boundary condition. They correspond to a constant temperature heat source, constant heat flow heat source, and constant convective heat source, respectively. Thus, this section analyzes the performance of the TPGS under the above three heat sources, which has specific guiding significance to the practical application of the system.

#### 3.2.1. Constant Temperature Heat Sources

**Figure 10:**
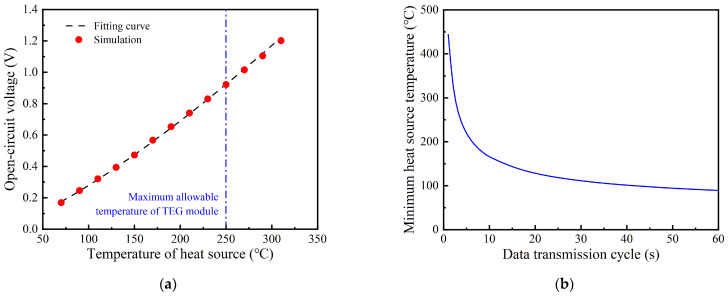
The performance of TPGS under constant temperature heat sources: (**a**) open−circuit voltage; (**b**) the minimum heat source temperature for powering wireless sensor.

When the heat source is abundant, the temperature of the heat source is almost unchanged because the heat absorbed by the thermoelectric self−powered system is much lower than that in the heat source. The Dirichlet boundary condition can reasonably predict the influence of this heat source on the system. The heat source temperature is increased from 70 °C to 310 °C with an interval of 20 °C. The open−circuit voltage increases rapidly in a power function with the increase in the heat source temperature, as shown in Figure 10a. Assuming the circuit efficiency of the system is 9.45%, the minimum temperature of the heat source for the wireless sensor with different data transmission cycles can be estimated, as shown in Figure 10b. The temperature of the heat source needs to achieve 111 °C to meet the power demand of the wireless sensor with a data transmission cycle of 30 s. Meanwhile, the heat source temperature cannot exceed 250 °C because of the maximum allowable temperature of the TEG module.

#### 3.2.2. Constant Heat Flow Heat Sources

**Figure 11:**
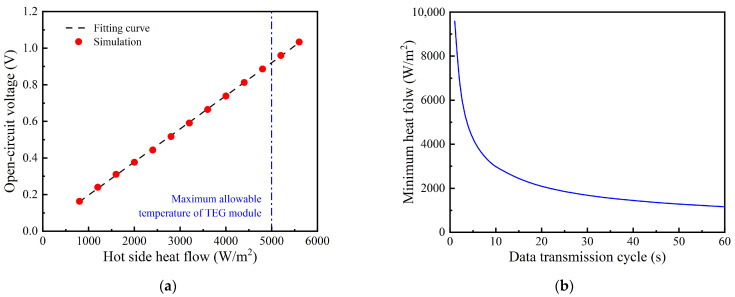
The performance of TPGS under constant heat flow heat sources: (**a**) open−circuit voltage; (**b**) the minimum heat flow for powering wireless sensor.

However, when the heat source is unabundant, the heat consumed by the TPGS cannot be neglected because it is not much smaller than the heat flow of the heat source. Thus, the Neumann boundary condition can reasonably foretell the impact of this heat source on the system. The heat flow of the heat source is increased from 800 W/m^2^ to 5600 W/m^2^ with an interval of 400 W/m^2^. The open−circuit voltage increases linearly with the increase in the heat source temperature, as shown in Figure 11a. Considering the circuit efficiency of the system is 9.45%, the minimum heat flow of the heat source for the wireless sensor with different data transmission cycles can be evaluated, as shown in Figure 11b. The heat flow of the heat source needs to obtain 1681 W/m^2^ to match the power demand of the wireless sensor with the data transmission cycle of 30 s. Simultaneously, the heat flow is limited to 5000 W/m^2^ because of the maximum allowable temperature of the TEG module.

#### 3.2.3. Constant Heat Convection Heat Sources

**Figure 12:**
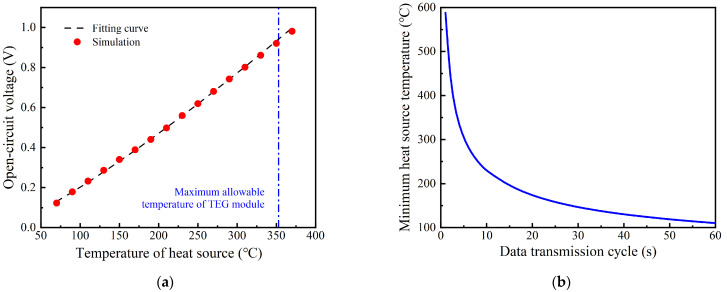
The performance of TPGS under constant heat convection heat sources: (**a**) open−circuit voltage; (**b**) the minimum heat source temperature for powering wireless sensor.

In the actual industrial production process, the waste heat is not only lost through walls but also exists in the exhaust gas. If the heat from the exhaust gas is expected to be used, fins are required on the hot side of the copper substrate to strengthen the heat exchange between the TPGS and the heat source. Thus, the Robin boundary condition is in accord with this condition and used to calculate the influence of this heat source on the system. In this section, a flat fin is added to the hot side of the copper substrate. The convective heat transfer coefficient of the flat fin is about 22 W/m^2^/K~35 W/m^2^/K for the exhaust gas velocity of 1 m/s. Considering that the fin area is two to three times larger than the substrate area, the equivalent convective heat transfer coefficient is set to 50 W/m^2^/K in this section. The heat source temperature is increased from 70 °C to 370 °C, with an interval of 20 °C. The open−circuit voltage increases rapidly in a power function with the increase in the heat source temperature, as shown in Figure 12a. Considering the circuit efficiency of the system is 9.45%, the minimum heat source temperature for the wireless sensor with different data transmission cycles can be evaluated, as shown in Figure 12b. The temperature of the heat source needs to achieve 146 °C to meet the power demand of the wireless sensor with the data transmission cycle of 30 s under the constant heat convection heat sources. Meanwhile, the heat source temperature cannot exceed 353 °C because of the maximum allowable temperature of the TEG module.

## 4. Experimental Analysis of Thermoelectric Self−Powered System

**Figure 13:**
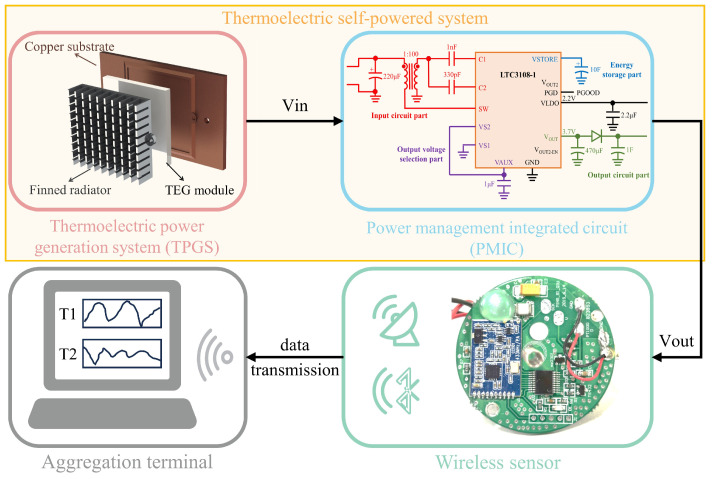
Block diagram of the thermoelectric self−powered sensor.

In this chapter, the total system of the thermoelectric self−powered sensor, including the thermoelectric power generation system, power management integrated circuit, wireless sensor, and aggregation terminal, was fabricated and tested under specific conditions. As shown in Figure 13, the thermoelectric self−powered system contains TPGS and PMIC. The power generated by the TPGS is collected and stored by the PMIC, and then provided to the wireless sensor. Then, the detailed actual performance of the thermoelectric self−powered system was investigated, and the economic benefit of this system was also analyzed to perceive its commercialization prospect. Thus, the performance and cost of the thermoelectric self−powered system are cleared through the experiments, which is of great significance in promoting the application of the self−powered wireless sensor.

### 4.1. Performance Test of Thermoelectric Self−Powered System

**Figure 14:**
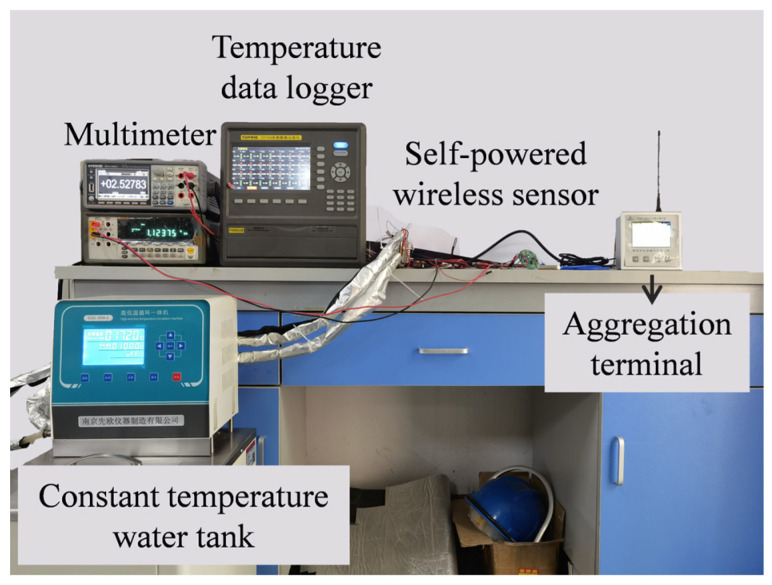
The test rig for testing the performance of the thermoelectric self−powered wireless sensor.

The test rig for the thermoelectric self−powered sensor wireless was built based on testing the open−circuit voltage of the self−powered system. The photo of this test rig is shown in Figure 14. A constant temperature water tank as the heat source was used to provide heat to the TPGS, and the details of the TPGS were described in Section 3.1. The circuit system was set up according to Figure 5 and connected between the TPGS and the wireless sensor. Furthermore, the temperatures at key locations were tested by T−type thermocouples (TT−T−40−SLE) and transmitted to a data logger (TOPRIE TP700). The data−receiving condition of the aggregation terminal is monitored to ensure the regular operation of the wireless sensor. The temperature of the heat source was maintained at 70 °C, and the wireless sensor was set to transmit data at 39.38 s intervals.

**Figure 15:**
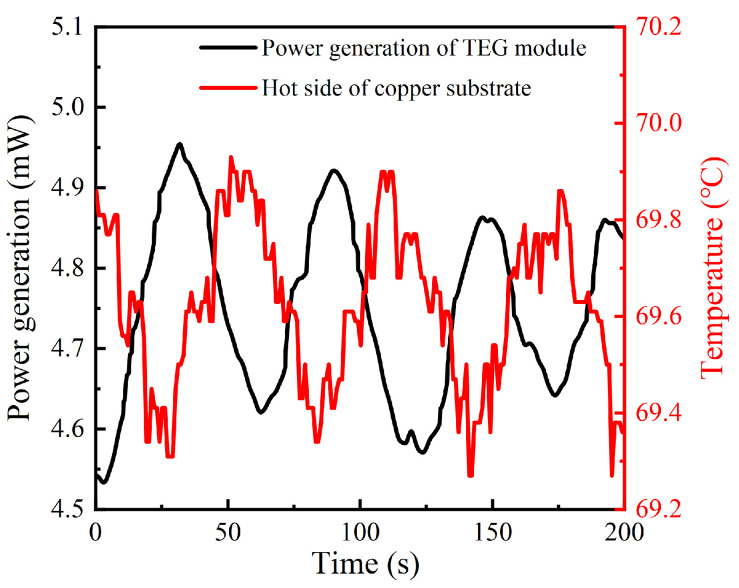
Variation of TEG’s power generation and hot side temperature of copper substrate with time.

Firstly, the power generation of the TEG module was also measured based on the test rig. A noteworthy phenomenon is that the power generation of the TEG module and the hot side temperature of the copper substrate is oscillating around a specific value when the thermoelectric self−powered system is stable, as shown in Figure 15. The shape of the curve is close to the shape of the sinusoidal curve. Meanwhile, the point−in−time of the peak power generation and the trough temperature are almost consistent, indicating an unavoidable delay between temperature and power generation. The delay time is about 36 s. According to Figure 15, the average power generation of the TEG module is 4.75 mW when the heat source temperature is 70 °C.

Then, the power consumption of the wireless sensor can be estimated according to the data tested in Section 2.1. When the data transmission cycle of the wireless sensor is set to 39.38 s, the average power consumption is 0.67 mW and the energy consumption is 0.026 J during one data transmission cycle.

**Figure 16:**
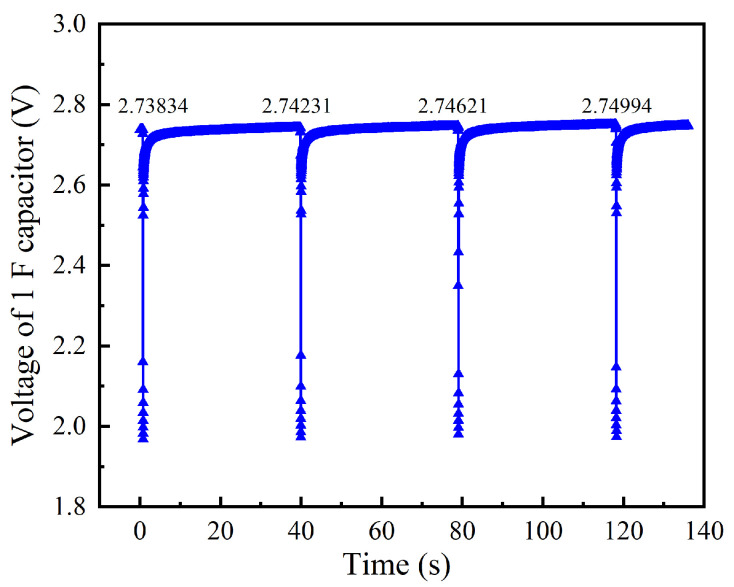
Variation of the 1 F capacitor voltage versus time.

Finally, the energy stored in the capacitor was also tested. The 1 F capacitor voltage is displayed in Figure 16. Taking the first data transmission cycle as an example, when the wireless sensor is running, the voltage of the 1 F capacitor drops instantaneously from 2.738 V to 1.973 V in 0.09 s and then bounces back to 2.621 V in 0.05 s. As the TEG module charges the capacitor, its voltage slowly retunes to 2.742 V under the dormant state. However, the voltage of another 10 F capacitor is almost unchanged, which means that no electrical energy is stored in that capacitor. Thus, the energy stored in the capacitor in each cycle can be obtained by the voltage change of 1 F capacitor, which is calculated by:

*W_s_* = 0.5 × *C*_1*F*_ × (*V*_2_^2^
*– V*_1_^2^) (3)

where *W_s_*, *C*_1*F*_, *V*_2_, and *V*_1_ represent the stored energy, 1 F capacitor’s capacitance, voltage at the end of each cycle, and the voltage at the beginning of each cycle, respectively. Therefore, as for the three complete data transmission cycles in Figure 16, the energy stored in the capacitor is 0.0109 J, 0.0107 J, and 0.0103 J, respectively. The average energy stored is 0.0106 J per cycle.

Moreover, although the capacitor’s voltage does not reach the voltage of the battery used in the commercial wireless sensor, the wireless sensor can still work, which can be confirmed by observing the aggregation terminal.

**Figure 17:**
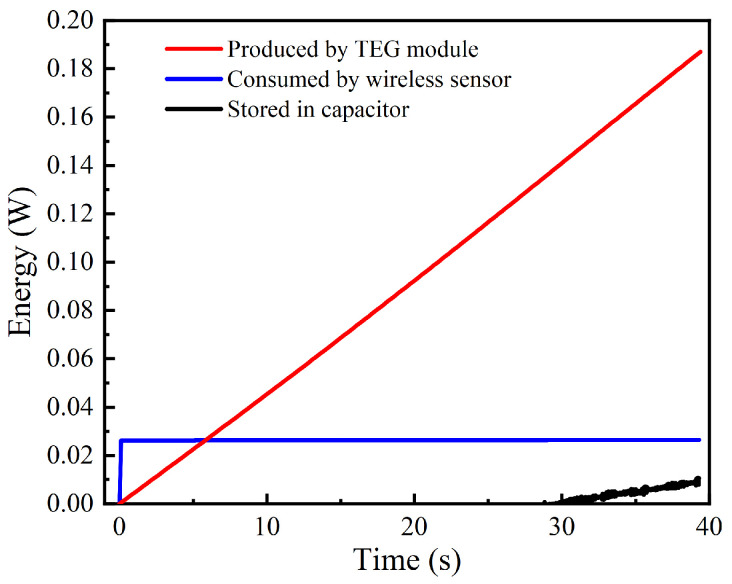
Energy produced or consumed by each part of the thermoelectric self−powered wireless sensor in a data transmission cycle.

Based on the above analysis, Figure 17 depicts the energy produced or consumed by each part of the thermoelectric self−powered wireless sensor in a data transmission cycle. The electricity generated by the TEG module increases linearly with time. The wireless sensor transmits data to the aggregation terminal at first and consumes much electricity stored in the capacity in advance. Then, it comes into the dormant state and consumes almost no energy. Accordingly, the charge stored in the capacitor does not exceed the initial charge until 30 s, which means the electricity stored by the capacitor in 30 s is only enough for the wireless sensor to transmit data once in this condition. In this data transmission cycle, the energy produced by the TEG module, consumed by the wireless, and stored in the capacitor are 0.187 J, 0.026 J, and 0.0106 J, respectively. Therefore, only 19.57% of electricity is used availably, and the rest is lost in the process of boost and storage. This is also consistent with the datasheet of LTC3108−1.

The capacitor can store the energy of 0.0106 J during a data transmission cycle, which accounts for 40.8% of the wireless sensor energy consumption of one cycle. It can be seen that the current system is already enough to support the regular operation of the wireless sensor when the heat source temperature is 70 °C. However, the circuit system is less efficient, so there is still ample space for optimization.

### 4.2. Economic Benefit Analysis

To analyze the commercial prospects of the thermoelectric self−powered system, the cost of this system must be considered. The expense of each part of the system is calculated, as shown in Table 4. It can be seen that the cost of one thermoelectric self−powered system is about EUR 9.1. At present, there already exist some batteries with long −life spans and high power capacities, such as the NL2160HP battery produced by NITECORE, which can support the wireless sensor for nearly 3.7 years, but this battery is expensive, costing at least EUR 21.6. The cost of the thermoelectric self−powered system in this paper is only 42% of the high−performance battery, while its life can be more than ten years. Thus, this system has a particular commercial application prospect. Moreover, this system utilizes industrial waste heat to power the wireless sensor, improving energy utilization and saving energy consumption. It is of great significance to promote the development of the IoT.

## 5. MPPT Optimization for the Output Power of TEG

Due to the fact that the cold side of the TPGS is cooled by natural convection and its hot side may be attached to a heat source surface at any temperature, the operating temperature and temperature difference of the TEG module in the TPGS will vary over time and with different application scenarios, leading to fluctuations in its open−circuit voltage and internal resistance. Moreover, the input resistance of the PMIC based on LTC3108−1 will vary with changes in the input voltage and the transformer turns ratio, with a range of 2 Ω to 10 Ω. The two factors make it difficult to match the external load resistance with the internal resistance of the TEG module, consequently hindering the TEG module from operating at its maximum output power point. Therefore, it is necessary to perform MPPT optimization on the TEG module to enhance its output power.

### 5.1. SPEIC Converter Simulation Model

**Figure 18:**
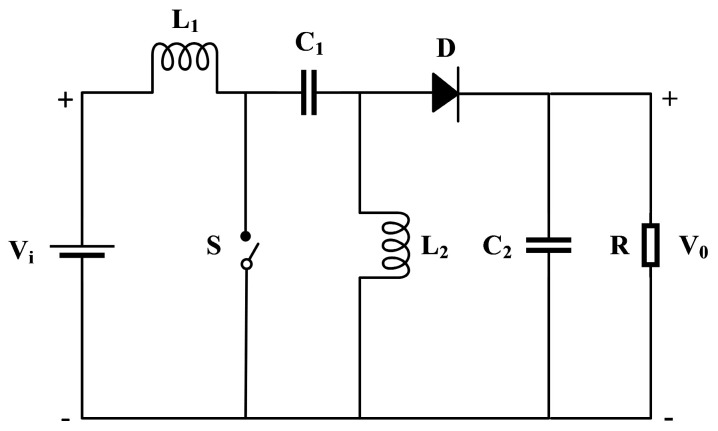
The circuit diagram of SEPIC converter.

The SEPIC converter can achieve boost and buck functions by adjusting the duty cycle of the main control switch (S), and it can achieve the same polarity of the output and input voltage through the capacitor (C_1_) on the main circuit. A standard SEPIC converter circuit configuration is illustrated in Figure 18, when the main control switch is completely closed, the output voltage is 0 V. Therefore, the SEPIC converter is utilized to conduct MPPT optimization research on the TEG module’s output power.

**Figure 19:**
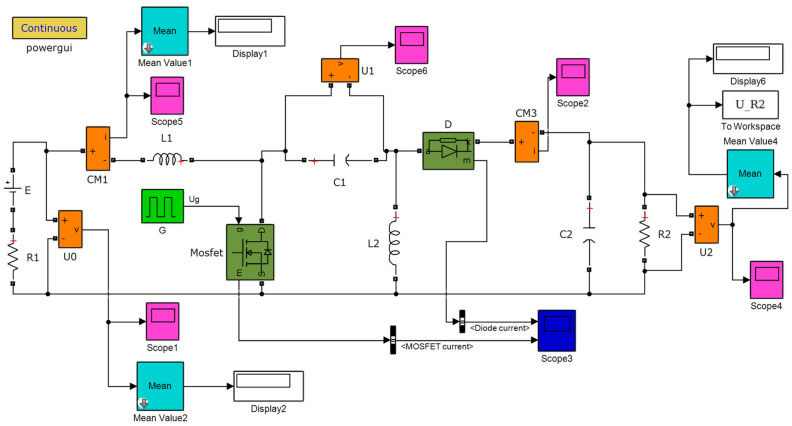
SEPIC converter simulation model.

Based on the principle of the SEPIC converter, a simulation model of the SEPIC converter was established in the Simulink software (version: R2017a), as shown in Figure 19. Related display modules were incorporated to obtain the voltage and current values at various points in the circuit, which facilitates studying the effect of MPPT optimization. In this model, the resistor R_1_ and the voltage source E, respectively, represent the internal resistance and the open−circuit voltage of the TEG module, which are connected in series to equivalently represent the TEG module. The resistor R2 represents the external load resistance connected to the TEG module, and the output power of the SEPIC converter equals the power consumption of resistor R2. The MOSFET, which stands for Metal−Oxide−Semiconductor Field−Effect Transistor, can adjust the duty cycle (*D*) through the signal sent by the pulse generator named G. The duty cycle is the ratio of the MOSFET’s on−time to the pulse period. Thus, the relationship between the output voltage (*U*_2_) and the input voltage (*U*_0_) of the SEPIC converter is expressed by:

*U*_2_ = *D*/(1 − *D*) × *U*_0_ (4)

### 5.2. Impact of MPPT Optimization on the TEG Output Power

**Figure 20:**
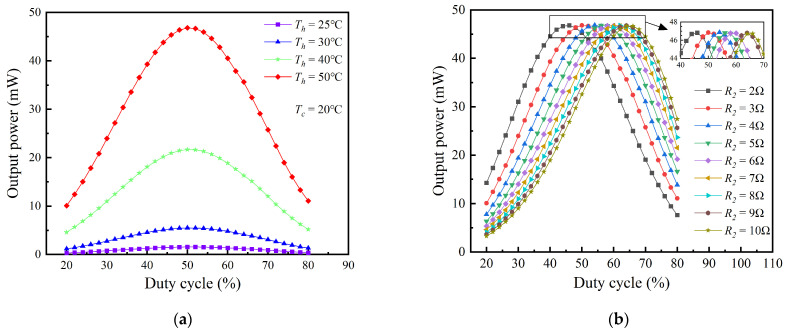
The variation of SEPIC converter’s output power with duty cycle under (**a**) different temperature differences of TEG, and (**b**) different external load resistance.

In practical applications, as for TPGS, the temperature of the heat source, the convective heat transfer coefficient of the cold side, and the variation of ambient temperature can all cause changes in the temperature gradient across the TEG module. As can be seen from Table 2, when the hot side temperatures of the TEG module are 25 °C, 30 °C, 40 °C, and 50 °C, and the cold side temperature is 20 °C, its internal resistance varies from 2.67 Ω to 2.86 Ω, and the open−circuit voltage changes from 0.13 V to 0.74 V. Therefore, the change in the output power of the TEG module through the SEPIC converter under different temperature differences was investigated, as shown in Figure 20a, with the external load resistance fixed at 3 Ω. It can be observed that under different temperature differences, the output power initially increases and then decreases with the increase in the duty cycle, and the difference in output power among different temperature differences also first increases and then decreases. At the maximum power point, the difference in output power among various temperature differences is the greatest, indicating that it is very necessary to conduct MPPT control for the TEG module. The duty cycle to achieve the maximum output power at different temperature differences is around 50%, and only a slight adjustment of the duty cycle is needed to obtain the maximum output power.

Moreover, in practical applications, the external load resistance connected to the TEG module is subject to variation. For instance, the input resistance of the PMIC based on LTC3108−1 fluctuates between 2 Ω and 10 Ω. Such variations can prevent the TEG module from operating at its maximum power point, thereby necessitating an investigation into the impact of external load resistance (*R*_2_) on output power. As illustrated in Figure 20b, when the TEG module’s hot side temperature is 50 °C and the cold side temperature is 20 °C, the output power initially increases and then decreases with the duty cycle under various *R*_2_, reaching a maximum value at a specific duty cycle. When the value of *R*_2_ changes, the corresponding maximum output power value remains almost constant, although the duty cycle corresponding to the maximum output power increases as the *R_2_* increases. As the value of *R*_2_ varies from 2 Ω to 10 Ω, the corresponding duty cycle to achieve maximum output power increases from 45% to 65%. This indicates that even with a mismatch in external load resistance, it is possible to maintain the TEG module’s maximum output power by adjusting the duty cycle.

**Figure 21:**
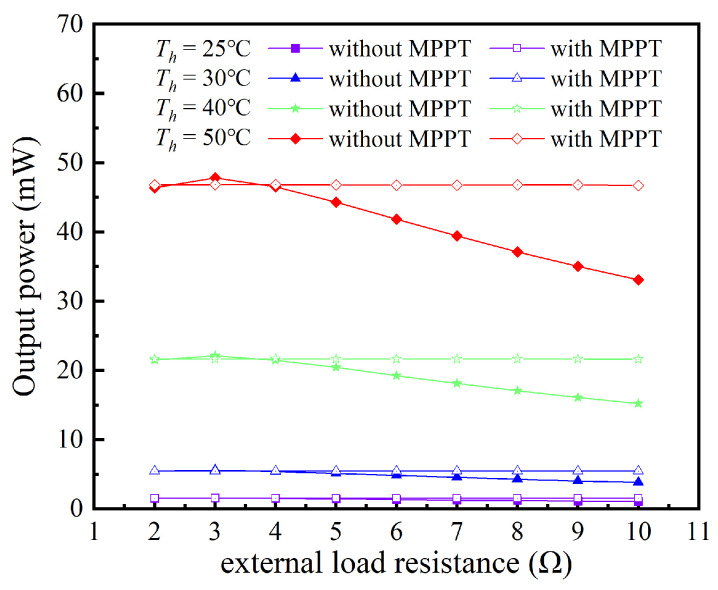
The impact of MPPT optimization on output power.

**Figure 22:**
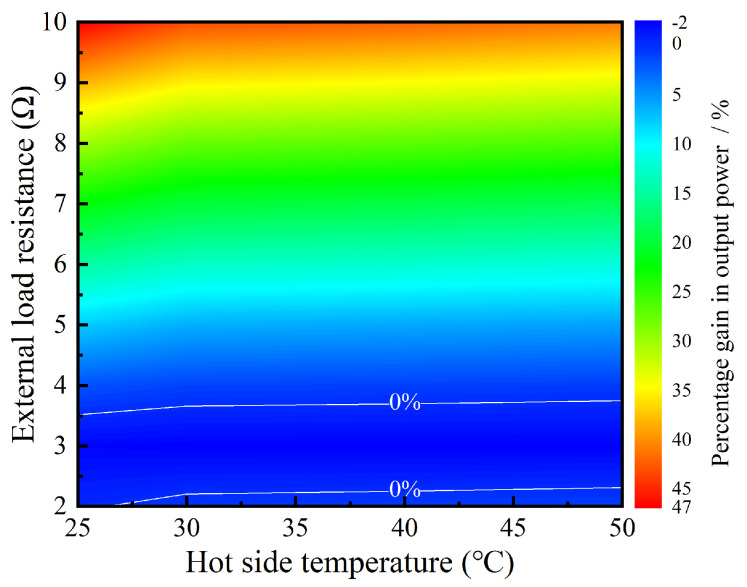
The percentage gain in output power under different conditions.

Based on the aforementioned analysis, it can be deduced that under various temperature differences across the TEG module and different external load resistances, the TEG module can operate at its maximum power point by adjusting the duty cycle of the SEPIC converter. In practical applications, a control circuit can be added to regulate the duty cycle of the SEPIC converter, thereby achieving the MPPT function for the TEG module. Figure 21 compares the impact of having or not having an MPPT function on the TEG module’s output power under different operating conditions. Without an MPPT function, the output power increases and then decreases with changes in the external load resistance, reaching a maximum when the load resistance is close to the TEG module’s internal resistance of approximately 3 Ω. With an MPPT function, the output power remains almost constant with changes in the external load resistance, and the TEG module operates very stably near its maximum power point. Additionally, the percentage gain in output power because of the MPPT function is depicted in Figure 22. When there is a significant difference between the external load resistance and the TEG module’s internal resistance, the enhancement in output power is quite evident. For instance, when the external load resistance is 10 Ω, the output power can increase by up to approximately 47%. However, when the external load resistance is close to the internal resistance, the output power may decrease slightly due to the circuit efficiency of the SEPIC converter, but the reduction percentage does not exceed 2%.

## 6. Conclusions

To solve the power consumption issue of the wireless sensor, a thermoelectric self−powered wireless sensor, which can be powered through environmental heat, was designed, fabricated, tested, and optimized. The main research contents and conclusions obtained in this paper are as follows:

1. The wireless sensor has two operational modes: a transmitting mode (0.08 s) and a dormant mode, with corresponding power of 326 mW and 5.45 μW, respectively. The TEG module can power the wireless sensor with the data transmission cycle of 13.2 s through the PMIC based on LTC3108−1 when the temperature difference is 12.5 °C. However, the circuit efficiency of the PMIC is only 9.45%.
2. A thermoelectric power generation system with a simple structure was designed, and the corresponding simulation model was established and verified by the experiments. The generation performance of this system is obtained under the Dirichlet, Neumann, and Robin boundary conditions. The minimum temperature of the heat source is 111 °C and 146 °C for the Dirichlet and Robin boundary conditions to meet the power demand of the wireless sensor with a data transmission cycle of 30 s. In the same power demand, the minimum heat flow is 1681 W/m^2^ for the Neumann boundary condition.
3. A thermoelectric self−powered wireless sensor was also fabricated and tested. When the heat source temperature is 70 °C, the self−powered wireless sensor can generally operate in the data transmission cycle of 39.38 s. The circuit efficiency of the PMIC is only 19.57%, and about 29% of the available electricity is stored in the capacitor. The cost of one thermoelectric self−powered system is about EUR 9.1, indicating that this system has a particular commercial application prospect.
4. The SEPIC converter model was established to conduct MPPT optimization for the TEG module. Under various temperature differences across the TEG module and different external load resistances, the TEG module can operate at its maximum power point by adjusting the duty cycle of the SEPIC converter. When external load resistance is 10 Ω, the output power can increase by up to approximately 47%.

## Figures and Tables

**Table 1:** Technical specifications of wireless sensor.

Technical Specification	Value	Unit
Frequency domain	433~434.79	MHz
Transmission distance	>800 (unobstructed)	m
Operating ambient temperature	−25~125	°C
Voltage of battery	3.6	V
Weight	170	g

**Table 2:** The resistance, open−circuit voltage, and maximum output power of TEG under various conditions.

*T_h_* (°C)	25	30	35	40	45	50
*R_in_* (Ω)	2.67	2.79	2.74	2.82	2.72	2.86
*U_oc_* (V)	0.13	0.25	0.38	0.50	0.62	0.74
*P_max_* (W)	0.0015	0.0057	0.0134	0.0225	0.0352	0.0478

**Table 3:** Design demand for the thermoelectric power generation system.

Data Transmission Cycle (s)	Average Power of Wireless Sensor (mW)	Power Needed From TPGS (mW)	Minimum Open−Circuit Voltage of TEG (V)
5	5.22	55.3	0.784
10	2.61	27.7	0.554
20	1.31	13.9	0.351
30	0.875	9.26	0.321
40	0.657	6.96	0.278
50	0.527	5.58	0.249
60	0.440	4.66	0.227

**Table 4:** Cost of the thermoelectric self−powered system.

Part	Material/Type	Price	Cost (EUR)
Substrate	Copper (C11000)	6.2~9.5 EUR/kg	0.9
TEG module	TEG1−127−1.4−1.6−250	EUR 3.1	3.1
Finned radiator	Aluminum (6061)	5.4~7.0 EUR/kg	0.1
Circuit system	LTC3108−1	EUR 3.8	3.8
Other	Shell, screw, wire, etc.	EUR 1.2	1.2

## Data Availability

The data that support the findings of this study are available from the corresponding author upon reasonable request.
